# Optimal hematoma volume cutoffs and efficacy of minimally invasive surgery for thalamic hemorrhage: a propensity score-matched analysis

**DOI:** 10.1186/s12883-026-04748-1

**Published:** 2026-02-27

**Authors:** Xu Zhao, Wanyu Ma, Wenying Zhang, Mingjie Deng, Likun Wang, Guofeng Wu, Siying Ren

**Affiliations:** 1 Emergency Department, the Affiliated Hospital of Guizhou Medical University, Guiyang, Guizhou Province 550004 China; 2 Key Lab of Acute Brain Injury and Function Repair, Guizhou Medical University, Guiyang, Guizhou Province 550025 China

**Keywords:** Thalamic hemorrhage, Minimally invasive surgery, Propensity score matching, Hematoma volume, Prognosis

## Abstract

**Background:**

Spontaneous thalamic hemorrhage (TH) is associated with substantial morbidity and mortality. The optimal hematoma volume (HV) thresholds for prognostic stratification and the comparative effectiveness of minimally invasive surgery (MIS) versus conservative treatment remain uncertain.

**Methods:**

We retrospectively analyzed consecutive patients with spontaneous TH admitted to a comprehensive stroke center *n* = 436 using a two-stage design. Stage 1: In the conservatively treated cohort *n* = 280, predictors of poor 90-day functional outcome (modified Rankin Scale [mRS] 3–6) were identified using multivariable logistic regression; a prognostic nomogram was constructed and validated. Receiver operating characteristic (ROC) analyses were used to determine HV cutoffs for predicting poor functional outcome and mortality. Stage 2: Patients with HV ≥ 7 mL were included in a comparative effectiveness analysis of MIS versus conservative treatment *n* = 208. Propensity score matching (PSM) was performed (1:1 nearest-neighbor without replacement; caliper = 0.2 SD of the logit of the propensity score) to balance baseline covariates, including age, sex, systolic blood pressure (SBP), Glasgow Coma Scale (GCS), National Institutes of Health Stroke Scale (NIHSS), intraventricular hemorrhage (IVH), Graeb score, and HV.

**Results:**

In conservatively treated patients *n* = 280, older age, higher SBP, and larger HV were independently associated with increased odds of poor 90-day outcome, whereas higher GCS was protective. The nomogram demonstrated good performance. ROC analyses identified 7 mL as the optimal HV cutoff for predicting poor functional outcome AUC = 0.86, and 13 mL for predicting mortality AUC = 0.81. After PSM in patients with HV ≥ 7 mL (*n* = 102; 51 matched pairs), MIS was associated with lower 90-day mortality compared with conservative treatment 9.8% vs. 29.4%, *P =* 0.01. However, the rate of functional independence did not differ significantly between groups 31.4% vs. 33.3%, *P* = 0.83.

**Conclusion:**

HV is a key prognostic marker in TH, with 7 mL and 13 mL serving as clinically relevant thresholds for functional impairment and mortality, respectively. Among patients with HV ≥ 7 mL, MIS was associated with reduced 90-day mortality but not improved functional independence compared with conservative treatment.

**Trial registration:**

This study adheres to the principles of the Declaration of Helsinki and is retrospectively registered in ClinicalTrials.gov (No. NCT05548530),Registration Date:08/31/2022.

**Supplementary Information:**

The online version contains supplementary material available at 10.1186/s12883-026-04748-1.

## Introduction

Spontaneous intracerebral hemorrhage (ICH) is a devastating cerebrovascular event with a heavy global burden. Worldwide, ICH accounts for approximately 10%–20% of all strokes, with incidences varying by region and ethnicity [1]. In Western populations, it represents about 10%–15% of stroke cases [2]. However, in Asian populations, the prevalence is notably higher, accounting for 20%–30% of all stroke cases, and is associated with significantly higher rates of disability and mortality compared to ischemic stroke [3]. The functional prognosis of ICH is influenced by the hemorrhage location, with lobar hemorrhage generally having a better outcome than subcortical hemorrhage. Among patients with subcortical hemorrhage, those with thalamic hemorrhage (TH) experience slower recovery and worse long-term functional prognosis [4–6].

TH accounts for 10%–15% of all ICH cases. TH affects deep brain structures, such as the ventricles, pallidum, and internal capsule, due to its unique anatomical location, with high mortality and disability rates [7–9]. Lateral TH and posterior TH are adjacent to the posterior limb of the internal capsule and can result in limb paralysis and poor outcome when the hematoma compresses the internal capsule [10].

Baseline hematoma volume (HV) is an independent predictor of hematoma expansion and poor outcome in patients with ICH [11, 12]. Therefore, the baseline HV of TH can serve as a valuable guide for clinical treatment and may benefit patients. Based on this viewpoint, we aimed to determine the specific HV cutoff values that affect the prognosis of patients with TH and compare the effects of different treatments, including minimally invasive surgery (MIS) (comprising stereotactic hematoma evacuation [SHE] and neuroendoscopic surgery [NES]), and conservative treatment, on prognosis.

## Methods

### Study population

The clinical baseline data of patients with spontaneous TH who were hospitalized at the Department of Emergency Neurology of the Affiliated Hospital of Guizhou Medical University were retrospectively and consecutively collected by searching the hospital information system from January 1, 2019, to September 30, 2025. The inclusion criteria were as follows: patients with spontaneous ICH, with the confirmed hemorrhage location in the thalamus based on clinical presentation, and head computed tomography (CT) on admission. The exclusion criteria were as follows: multiple ICH; TH due to intracranial tumor, aneurysm, trauma, post-infarction hemorrhagic transformation, or other lesions; TH due to coagulation dysfunction, substitution therapy, or anticoagulant medications; age > 80 years; admission time > 48 h; history of prior stroke; and baseline data or follow-up information not fully documented or available.

### Therapeutic modalities

#### Conservative treatments

Pharmacotherapy in internal medicine was conducted according to the 2022 Guideline for the Management of Patients With Spontaneous ICH: A Guideline From the American Heart Association/American Stroke Association [13]. The general treatment required constant attention to ensure a high level of care and monitor patient’s vital signs. Patients were instructed to stay in bed continuously, use supplemental oxygen, avoid emotional excitement, and go to the bathroom regularly. Internal medicine treatment involved hemostasis, blood pressure control to prevent rebleeding, blood glucose control, body temperature control, anticonvulsants, infection prevention, and dehydration to reduce intracranial pressure. Multiple systemic complications, such as cardiac insufficiency, cardiac arrhythmia, renal impairment, and gastrointestinal bleeding, were actively managed.

#### Surgical treatments

**Fig. 1:**
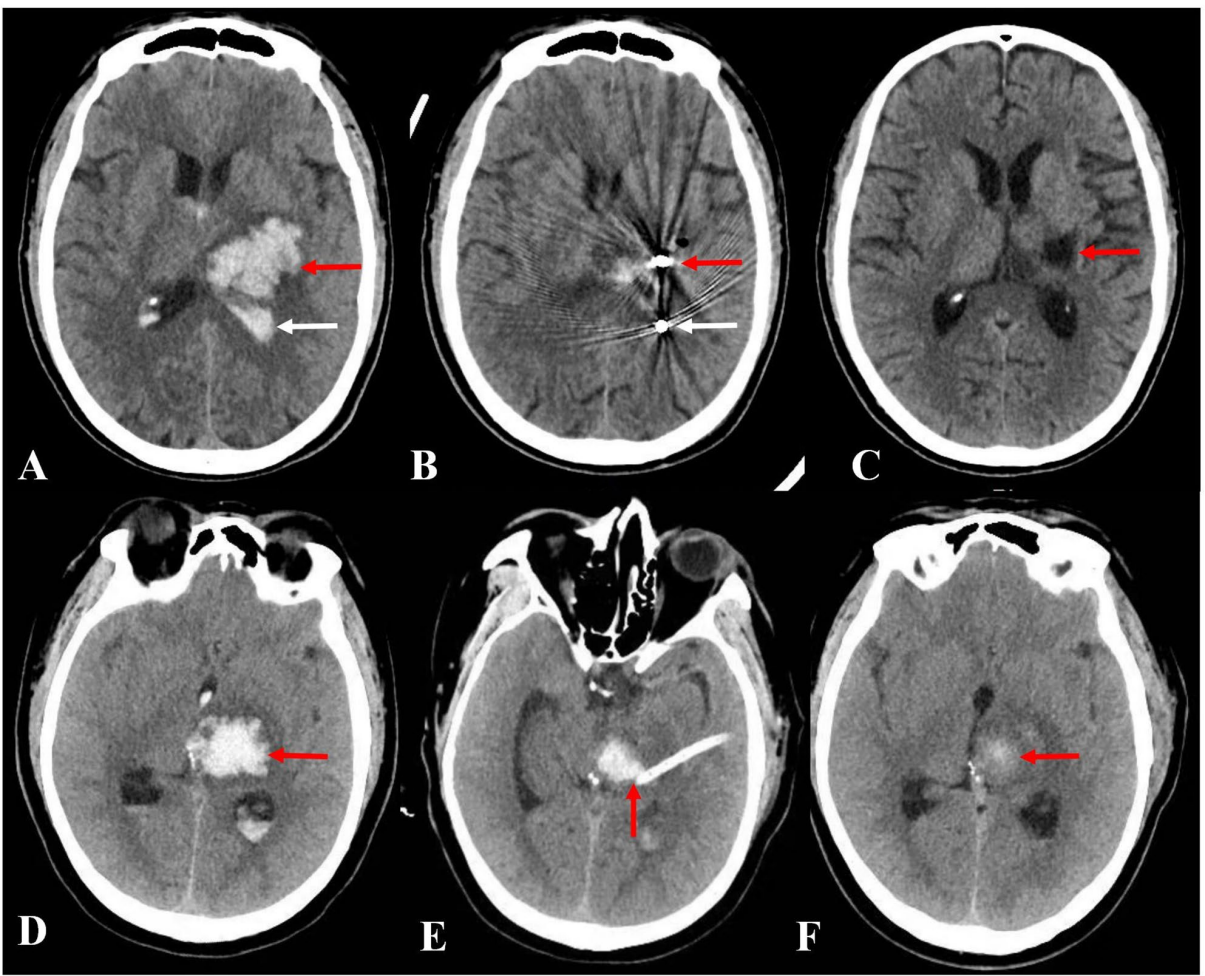
MIS treatment for patient with thalamic hemorrhage. (Figure ABC shows SHE treatment for thalamic hemorrhage. **A** Preoperative head CT scan, the red arrow denotes a thalamic hematoma, the white arrow showes hemorrhage within the inferior horn of the left lateral ventricle. **B** A head CT scan was conducted on the first day after SHE, the red arrow indicates the puncture needle in the thalamic hematoma, the white arrow indicates the puncture needle in the inferior horn of the left lateral ventricle. **C** A head CT scan was performed on the 14th day after SHE, the red arrow displays thalamic malacia. Figure DEF shows NES treatment for thalamic hemorrhage. **D** Preoperative head CT scan, the red arrow indicated the thalamic hematoma. **E** A head CT scan was performed on the first day after NES, the arrow indicates the drainage tube. **F** A head CT scan was performed on the 10th day after NES, the arrow shows residual hematoma)

The choice of therapeutic modality followed a case-by-case assessment based on current guidelines. Decisions were made through a consensus process by a multidisciplinary team, including two senior neurosurgeons and a neurologist. Key determinants included the patient’s clinical status (GCS score), imaging characteristics HV, location, and mass effect), and institutional resources. While there was no strict randomization protocol due to the retrospective nature of the study, surgery was generally recommended for patients with significant mass effect or progressive neurological deterioration, subject to informed consent and family preference.Two representative cases of MIS treatment for thalamic hemorrhage is shown in Fig. 1.

### Data collection and prognostic assessment indicators

The demographic and clinical information of the patients included age, sex, unhealthy lifestyle habits (e.g., history of smoking, alcohol consumption), prior medical conditions, and medication usage. Other data on admission included blood pressure, Glasgow Coma Scale (GCS) score [14], National Institutes of Health Stroke Scale (NIHSS) score [15], time from symptom onset to baseline CT scan, HV, whether the hematoma broke into the ventricle, Graeb score [16], and whether obstructive hydrocephalus was present. Clinical prognosis was assessed at 90 days after admission using the mRS. Since follow-up was conducted via telephone, to minimize subjectivity and ensure accuracy, a standardized structured interview algorithm for modified Rankin Scale (mRS) was rigorously employed. All assessments were performed by trained neurology residents who were blinded to the initial treatment allocation. For cases with ambiguous descriptions of functional independence, detailed inquiries regarding daily living activities (e.g., walking without assistance, attending to bodily needs) were made, and the final scoring was adjudicated by a senior neurologist [17]. A mRS score of 0–2 was defined as a good outcome, while a score of 3–6 was considered a poor outcome [18, 19].

### Calculation of HV

The HV was measured using the following formula: $\left[\mathrm{HV}\,\left(\mathrm{mL}\right)=1/2\times\mathrm{length}\,\left(\mathrm{cm}\right)\times\mathrm{width}\,\left(\mathrm{cm}\right)\times\mathrm{height}\,\left(\mathrm{cm}\right)\right]$ [20]. The measurements were independently evaluated and calculated by two neuroimaging experts and a neurologist. Any discrepancies were resolved through discussion until consensus was reached.

### Statistical analysis

Statistical analyses were performed using SPSS version 27.0 (IBM Corp., Armonk, NY, USA) and R (RStudio version 4.4.0). Categorical variables are presented as n (%). Continuous variables are presented as mean ± standard deviation (SD) for normally distributed data or median (interquartile range [IQR]) for non-normally distributed data. Between-group comparisons were performed using the chi-square test or Fisher’s exact test for categorical variables, and Student’s t-test or the Mann–Whitney U test for continuous variables, as appropriate. For comparisons among more than two groups, analysis of variance (ANOVA) or the Kruskal–Wallis test was used. All tests were two-sided, and *P* < 0.05 was considered statistically significant.

Univariate logistic regression analyses were first performed to screen candidate predictors of poor 3-month outcome (mRS 3–6). Variables with *P* < 0.10 in univariate analysis and/or clinical relevance were considered for multivariable logistic regression. Multicollinearity was assessed, and because NIHSS showed collinearity with GCS, NIHSS was excluded and GCS was retained in the final multivariable model. Based on the final multivariable model, a prognostic nomogram was constructed to facilitate individualized risk prediction, and its performance was evaluated using discrimination and calibration analyses, supplemented by decision curve analysis to assess clinical utility.

Receiver operating characteristic (ROC) curve analysis was used to evaluate the prognostic value of HV for functional outcome (good outcome: mRS 0–2; poor outcome: mRS 3–6) and 90-day mortality. The optimal cutoff values were determined using the Youden index (maximum of sensitivity + specificity − 1), and the corresponding area under the curve (AUC), sensitivity, and specificity were reported.

For the comparative effectiveness analysis of MIS versus conservative treatment, propensity score matching (PSM) was conducted in patients with HV ≥ 7 mL. Propensity scores were estimated using a logistic regression model including the following baseline covariates: age, sex, GCS, NIHSS, HV, Graeb score, IVH, and SBP. Patients were matched 1:1 using nearest-neighbor matching without replacement with a caliper width of 0.2 of the standard deviation of the logit of the propensity score. Covariate balance before and after matching was assessed using standardized mean differences (SMDs), with lower SMDs indicating improved balance. Outcomes were then compared between the matched groups.

## Results

**Fig. 2:**
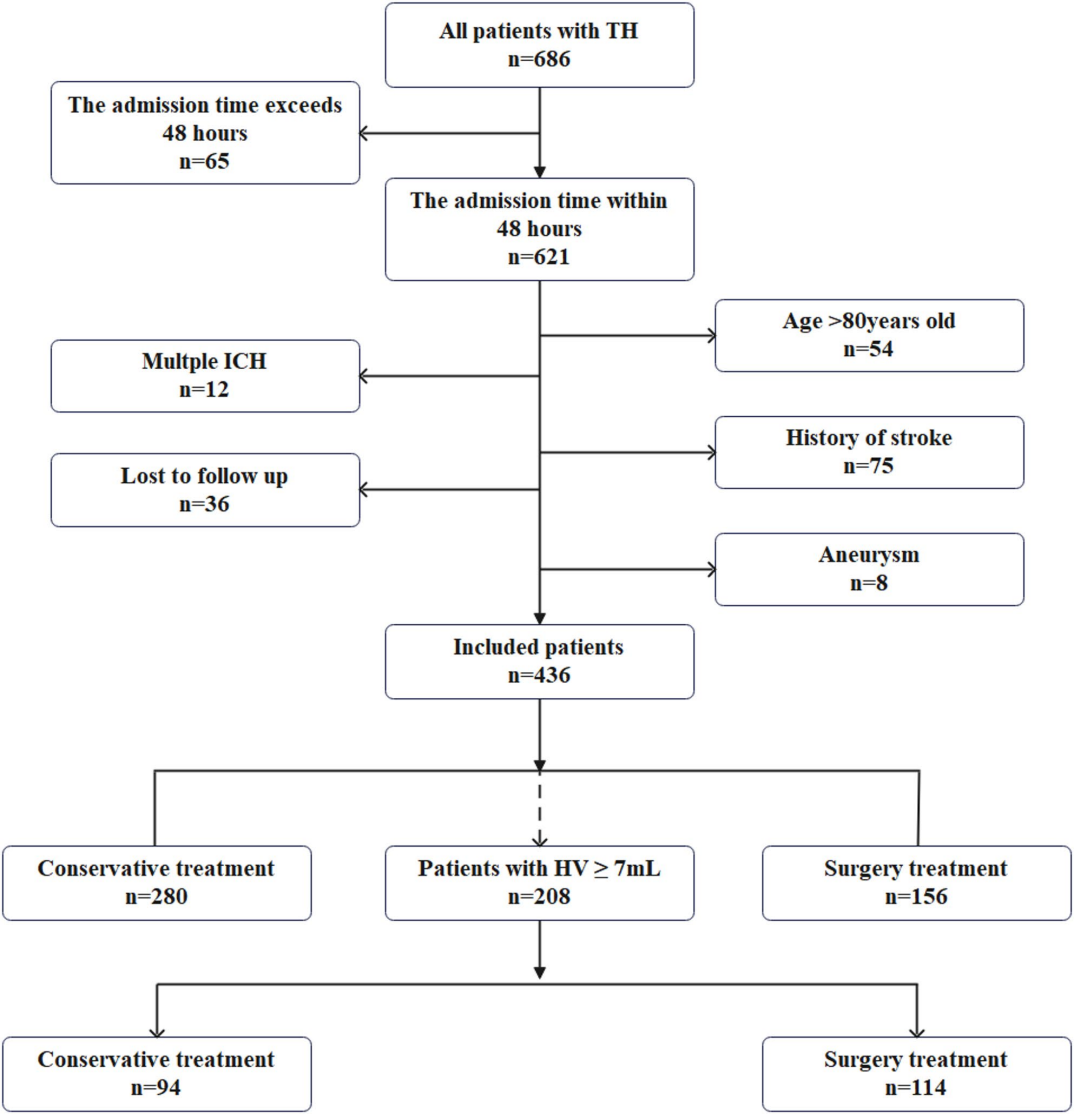
The flowchart of the participants selection. The diagram illustrates the patient inclusion and exclusion process, grouping, and subgroup analysis for hematoma volume (HV) ≥ 7 mL

During the study, 686 patients met the inclusion criteria. After applying the exclusion criteria, 436 patients were included in the final analysis (Fig. 2).

### Baseline characteristics and prognostic factors

A total of 280 patients with TH were included in the prognostic analysis (Table 1). At 3-month follow-up, 200 patients (71.4%) achieved a good outcome and 80 patients (28.6%) had a poor outcome. Patients with poor outcome were older and had higher admission SBP, lower GCS scores, higher NIHSS scores, larger HV, more frequent IVH, and higher Graeb scores than those with good outcome (Table 1).

**Table 1:** Baseline characteristics of the conservative treatment group stratified by 3-month functional outcome

Variables	Total (*n* = 280)	Good (*n* = 200)	Poor (*n* = 80)	*P*
Demographic				
Age (y), Mean ± SD	62.09 ± 9.67	60.98 ± 9.69	64.88 ± 9.09	0.002
Male Sex, n (%)	177 (63.21)	133 (66.50)	44 (55.00)	0.07
Medical history				
Smoking history, n (%)	112 (40.00)	80 (40.00)	32 (40.00)	1.00
Alcohol consumption, n (%)	102 (36.43)	71 (35.50)	31 (38.75)	0.61
Hypertension, n (%)	225 (80.36)	160 (80.00)	65 (81.25)	0.81
Diabetes, n(%)	32 (11.43)	22 (11.00)	10 (12.50)	0.72
Admission data				
SBP, mmHg(mean, SD)	169.82 ± 27.67	166.22 ± 26.05	178.82 ± 29.66	<0.001
DBP, mmHg(mean, SD)	100.67 ± 17.11	100.16 ± 16.81	101.96 ± 17.86	0.43
GCS (median, IQR)	14.50 (13.00, 15.00)	15.00(14.00, 15.00)	12.00(10.00, 14.00)	<0.001
NIHSS (median, IQR)	6.00 (3.00, 12.00)	4.00(2.00, 8.00)	14.50 (11.00, 21.25)	<0.001
Imaging characteristics				
HV, (mean, SD)	6.43 ± 6.04	4.33 ± 3.03	11.69 ± 8.17	<0.001
IVH, n(%)	167 (59.64)	105 (52.50)	62 (77.50)	<0.001
Graeb score (median, IQR)	2.00 (0, 3.00)	1.00(0, 2.00)	3.00(2.00,5.00)	<0.001

*TH* Thalamic hemorrhage, *SD* Standard deviation, *IQR* Inter-quartile range, *NIHSS* National Institutes of Health Stroke Scale, *GCS* Glasgow Coma Scale, *mRS* Modified Rankin Scale, *HV* Hematoma volume, *SBP* Systolic blood pressure, *DBP* Diastolic blood pressure, *IVH* Intraventricular hemorrhage, *y* year, *n* samples

*P *value < 0.05 was considered statistically significant

Univariate logistic regression identified age, SBP, NIHSS, GCS, IVH, HV, and Graeb score as significant predictors of poor 3-month outcome (Supplementary Table 1). Given collinearity between NIHSS and GCS, NIHSS was excluded from the multivariable model (Supplementary Table 2). In multivariable logistic regression, age (OR = 1.08, 95% CI: 1.03–1.12; *P* < 0.001), SBP (OR = 1.014, 95% CI: 1.01–1.03; *P* = 0.03), and HV (OR = 1.42, 95% CI: 1.26–1.59; *P* < 0.001) remained independent risk factors for poor outcome, whereas higher GCS was independently protective (OR = 0.78, 95% CI: 0.67–0.92; *P* = 0.003) (Supplementary Table 2).

### Determination of optimal cutoff values

**Fig. 3:**
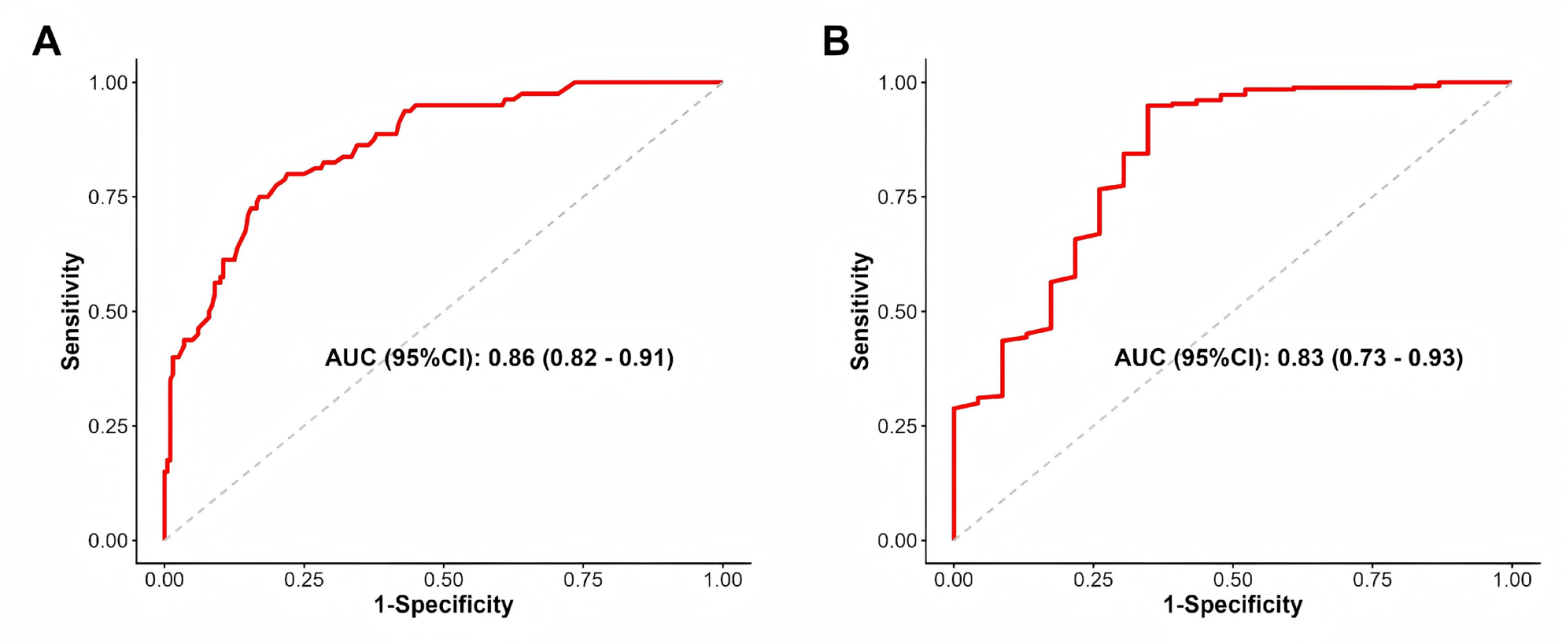
ROC Curve Analysis and Model Comparison. **A** ROC curve for predicting poor functional outcome (AUC = 0.86, optimal cutoff HV < 7.0 mL). **B** ROC curve for predicting 90-day mortality (AUC = 0.81, optimal cutoff HV > 13.0 mL)

ROC curve analysis was performed to evaluate the prognostic performance of HV for 90-day outcomes. For predicting poor functional outcome, the AUC was 0.86, with an optimal HV cutoff of 7.0 mL (sensitivity: 0.75, specificity: 0.83) (Fig. 3A). Based on this finding, HV ≥ 7 mL was defined as the threshold indicating a high-risk subgroup and was used as the criterion for subsequent comparative analyses. In addition, the optimal cutoff for predicting 90-day mortality was 13.0 mL (AUC = 0.81) (Fig. 3B) (Supplementary Table 3).

### Construction and validation of a prognostic nomogram

**Fig. 4:**
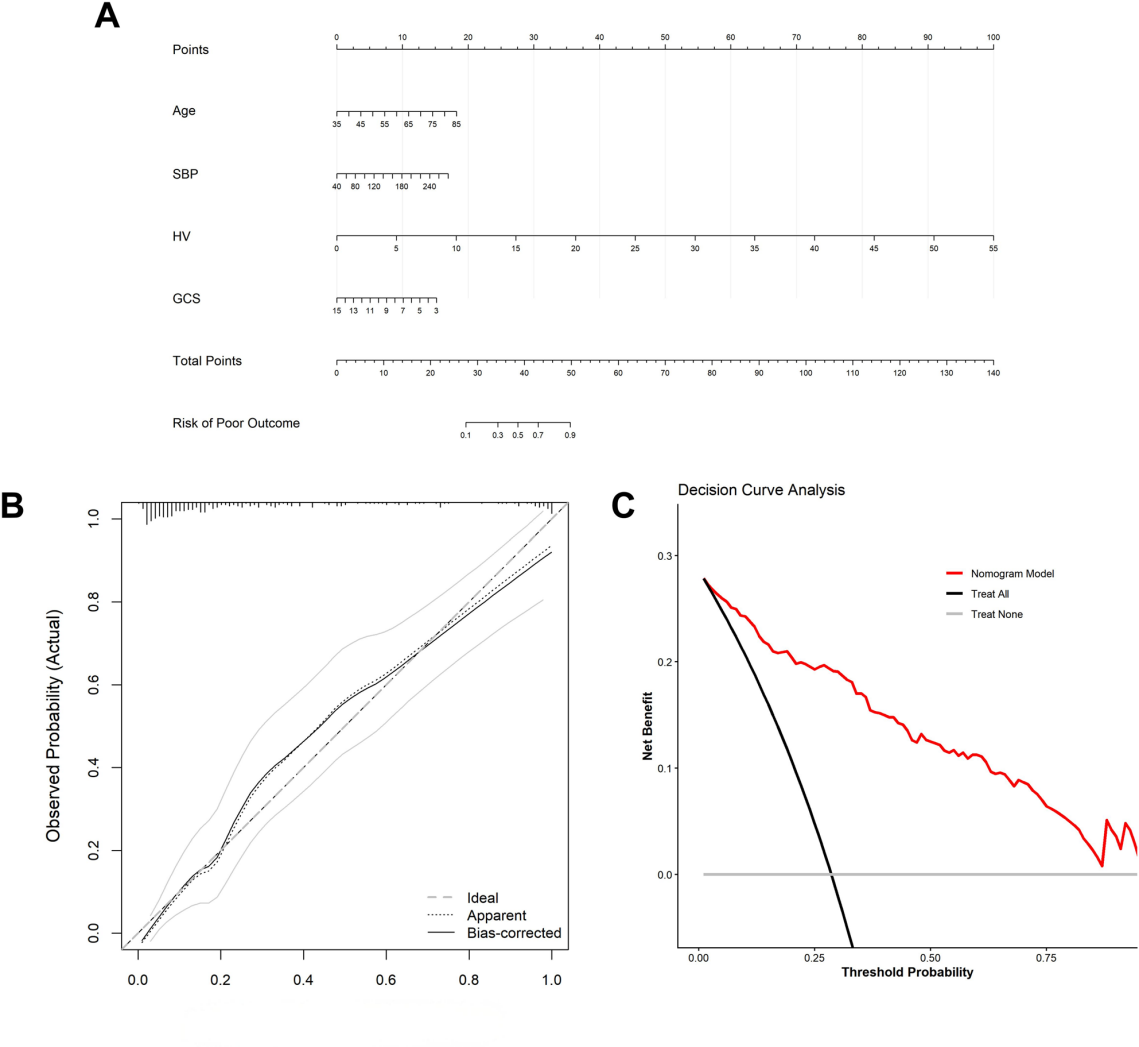
Construction and validation of the prognostic nomogram. **A** Nomogram for predicting the risk of poor 3-month outcome in patients with thalamic hemorrhage. Predictors were derived from the multivariable logistic regression model (Supplementary Table 2) and include Age, SBP, HV, and GCS. **B** Calibration curve of the nomogram. The x-axis represents the predicted probability, and the y-axis represents the actual observed probability. The diagonal dotted line represents perfect prediction; the solid line represents the performance of the nomogram (bias-corrected), indicating good consistency. **C** Decision Curve Analysis (DCA) for the nomogram. The red line represents the nomogram model, which shows a higher net benefit across a wide range of threshold probabilities compared to the “Treat All” (black line) and “Treat None” (grey line) strategies

To facilitate individualized prediction of 90-day functional outcome, a prognostic nomogram was constructed based on the independent predictors identified in the multivariable logistic regression model (Supplementary Table 2): Age, SBP, HV, and GCS (Fig. 4A). In the nomogram, each predictor is assigned a score on the point scale; the sum of these scores corresponds to an estimated probability of poor 3-month outcome.

The model performance was further assessed using calibration and decision curve analyses. The calibration curve (Fig. 4B) demonstrated good agreement between the nomogram-predicted probabilities and the observed outcomes. Decision curve analysis (Fig. 4C) indicated that the nomogram provided a net clinical benefit across a range of threshold probabilities compared with the default strategies of treating all or treating none.

### Comparison of therapeutic modalities for patients with HV ≥ 7 mL (before PSM)

Based on the ROC-derived threshold, 208 patients with HV ≥ 7 mL were included to compare therapeutic outcomes between MIS and conservative treatment (Table 2). In the MIS group, the median time from admission to surgery was 8 h (IQR: 7–11.5). All surgical interventions were performed on an emergency basis following admission.Before PSM, patients in the MIS group (*n* = 114) were younger but presented with more severe neurological deficits (lower GCS and higher NIHSS scores) and larger HV than those in the conservative group (*n* = 94) (all *P* < 0.05). In the unmatched cohort, 90-day mortality did not differ significantly between the two groups (18.09% vs. 21.05%, *P* = 0.59).

**Table 2:** Comparison of different therapeutic modalities for≥ 7mL TH (Before PSM)

Variables	Total *n* = 208	Conservative treatment *N* = 94	MIS *N* = 114	*P* value
Demographic				
Age, y(mean, SD)	59.88 ± 10.21	62.32 ± 9.34	57.86 ± 10.49	0.002
Male Sex, n(%)	144 (69.23)	58 (61.70)	86 (75.44)	0.03
Medical history				
Smoking history, n(%)	102 (49.04)	42 (44.68)	60 (52.63)	0.25
Drinking history, n(%)	95 (45.67)	40 (42.55)	55 (48.25)	0.41
Hypertension, n(%)	166 (79.81)	79 (84.04)	87 (76.32)	0.16
Diabetes, n(%)	21 (10.1)	9 (9.57)	12 (10.53)	0.82
Admission data				
SBP, mmHg(mean, SD)	175.55 ± 28.16	174.12 ± 28.26	176.73 ± 28.15	0.51
DBP, mmHg(mean, SD)	104.75 ± 18.71	102.53 ± 16.79	106.58 ± 20.04	0.12
GCS (median, IQR)	10.00 (7.00, 14.00)	13.00 (10.00, 15.00)	9.00 (6.00, 12.00)	<0.001
NIHSS (median, IQR)	18.00 (10.00, 33.25)	12.50 (8.25, 20.00)	24.00 (15.00, 35.75)	<0.001
Imaging characteristics				
HV, mL(mean, SD)	11.90 (9.17, 17.20)	10.00 (8.12, 13.97)	14.20 (10.72, 19.15)	<0.001
IVH, n(%)	189 (90.87)	78 (82.98)	111 (97.37)	<0.001
Graeb score (median, IQR)	4.00 (2.00, 6.00)	2.50 (2.00, 5.00)	5.00 (4.00, 7.00)	<0.001
Prognosis				
Good, n(%)	63 (30.29)	34 (36.17)	29 (25.44)	0.09
In-hospital Mortality, n (%)	7 (3.37)	4 (4.26)	3(2.63)	0.07
Mortality, n(%)	41 (19.71)	17 (18.09)	24 (21.05)	0.59

*PSM* Propensity Score Matching, *TH* Thalamic hemorrhage, *SD* Standard deviation, *IQR* Inter-quartile range, *NIHSS* National Institutes of Health Stroke Scale, *GCS* Glasgow Coma Scale, *IVH* Intraventricular hemorrhage, *SBP* Systolic blood pressure, *DBP* Diastolic blood pressure, *HV* Hematoma volume, *MIS* Minimally invasive surgery, *n* samples

*P* value < 0.05 was considered statistically significant

### Efficacy of MIS versus conservative treatment after PSM

After 1:1 PSM, 51 matched pairs (*n* = 102) were generated, with improved balance in baseline covariates (Table 3; covariate balance assessed by SMD, see Supplementary Fig. 1). In the matched cohort, MIS was associated with a lower 90-day mortality rate compared with conservative treatment (9.80% vs. 29.41%, *P* = 0.01). However, the proportion of patients achieving functional independence was not significantly different between groups (31.37% vs. 33.33%, *P* = 0.83). In-hospital mortality was 0% in the MIS group versus 5.9% in the conservative group, although this difference was not statistically significant (*P* = 0.24).

**Table 3:** Comparison of different therapeutic modalities for ≥ 7mL TH after PSM

Variables	Total *n* = 102	Conservative treatment *N* = 51	MIS *N* = 51	*P* value
Demographic				
Age, y(mean, SD)	60.41 ± 9.87	61.10 ± 8.81	59.73 ± 10.87	0.49
Male Sex, n(%)	68 (66.67)	33 (64.71)	35 (68.63)	0.67
Medical history				
Smoking history, n(%)	50 (49.02)	25 (49.02)	25 (49.02)	1
Drinking history, n(%)	45 (44.12)	22 (43.14)	23 (45.10)	0.84
Hypertension, n(%)	84 (82.35)	42 (82.35)	42 (82.35)	1
Diabetes, n(%)	12 (11.76)	7 (13.73)	5 (9.80)	0.54
Admission data				
SBP, mmHg(mean, SD)	172.82 ± 28.32	176.37 ± 30.19	169.27 ± 26.12	0.21
DBP, mmHg(mean, SD)	102.17 ± 16.39	102.78 ± 17.55	101.55 ± 15.30	0.71
GCS (median, IQR)	11.50 (9.00, 14.00)	11.00 (9.00, 14.00)	12.00 (9.00, 13.00)	0.78
NIHSS (median, IQR)	14.50 (10.00, 23.00)	14.00 (10.00, 23.50)	15.00 (10.00, 22.50)	0.89
Imaging characteristics				
HV, mL(mean, SD)	12.25 (9.45, 16.95)	10.90 (9.00, 16.65)	13.00 (10.40, 17.10)	0.17
IVH, n(%)	95 (93.14)	47 (92.16)	48 (94.12)	1
Graeb score (median, IQR)	4.00 (2.00, 5.00)	4.00 (2.00, 5.00)	4.00 (2.00, 5.00)	0.75
Prognosis				
Good, n(%)	33 (32.35)	17 (33.33)	16 (31.37)	0.83
In-hospital Mortality, n (%)	3 (2.94)	3 (5.88)	0	0.24
Mortality, n(%)	20 (19.61)	15 (29.41)	5 (9.80)	0.01

*PSM* Propensity Score Matching, *TH* Thalamic hemorrhage, *SD* Standard deviation, *IQR* Inter-quartile range, *NIHSS* National Institutes of Health Stroke Scale, *GCS* Glasgow Coma Scale, *IVH* Intraventricular hemorrhage, *SBP* Systolic blood pressure, *DBP* Diastolic blood pressure, *HV* Hematoma volume, *MIS* Minimally invasive surgery, *n* samples

*P *value < 0.05 was considered statistically significant

## Discussion

Previous studies have established that HV is an independent predictor of prognosis in patients with TH [11], a finding consistent with our results. Due to the thalamus’s critical location adjacent to the posterior limb of the internal capsule and the lateral ventricles, larger hematomas are more likely to disrupt the corticospinal tract, resulting in severe disability. Therefore, stratifying treatment strategies based on HV is essential for optimizing outcomes. Our ROC analysis identified an HV cutoff of < 7 mL for predicting a good functional outcome (AUC = 0.86, Sensitivity 0.75, Specificity 0.83) and > 13 mL for predicting mortality (AUC = 0.81). These thresholds refine those reported in earlier studies. For instance, Audrey et al., integrating data from ERICH and ATACH-II, proposed an 8 mL threshold for poor prognosis (AUC 0.79) [21], while a recent region-specific analysis identified 6.5 mL as the cut-off for good prognosis and 10.5 mL for high mortality risk [22]. Our findings, derived from a specific TH cohort, align closely with these values but demonstrated superior predictive performance (AUC 0.86 vs. 0.81), providing robust evidence for using 7 mL as a critical decision-making threshold.

The surgical management of TH remains a subject of intense debate. While traditional craniotomy is often associated with significant trauma due to the deep location of the thalamus, MIS offers a theoretical advantage by reducing tissue injury. Our study is highly relevant in the context of the recently published ENRICH trial [23], a landmark multicenter randomized clinical trial evaluating parafascicular surgery for intracerebral hemorrhage. The ENRICH trial demonstrated that early MIS significantly improved functional outcomes in lobar hemorrhages but failed to show a statistical benefit for hemorrhages located in the basal ganglia or thalamus (anterior basal ganglia stratum). This discrepancy highlights a critical knowledge gap: deeper lesions, such as TH, may require different selection criteria or surgical nuances compared to lobar bleeds. Our study directly addresses this limitation by focusing exclusively on TH and utilizing a volume-specific threshold (≥ 7 mL) for intervention. Unlike the ENRICH trial, which primarily focused on functional utility (mRS), our PSM analysis revealed that while MIS did not significantly improve the rate of good functional outcome (mRS 0–2) in patients with HV ≥ 7 mL, it was associated with a lower the mortality rate (9.8% vs. 29.4%, *P* = 0.013). This suggests that for deep-seated thalamic hemorrhages, the primary benefit of MIS may be “associated with improved survival outcomes” rather than “function-restoring” in the short term. By mitigating mass effect and minimizing secondary injury from blood breakdown products in the thalamus and ventricles, MIS may prevent fatal herniation or severe metabolic derangement, even if the primary damage to the internal capsule prevents immediate functional recovery. This distinction is crucial for patient counseling and refining the inclusion criteria for future trials targeting deep brain hemorrhages.

In contrast to the MISTIE III trial, which employed catheter-based aspiration combined with rt-PA and found no significant functional benefit compared to conservative treatment, our study utilized predominantly endoscopic and stereotactic evacuation techniques [24]. These active evacuation methods allow for more immediate hematoma reduction compared to passive drainage. Our results indicate that for massive TH, intervention is critical for survival. The lack of functional improvement in our surgical group, despite improved survival, is consistent with the “disability paradox” reported in stroke trials, in which interventions may be associated with improved survival outcomes but can result in a greater number of survivors living with severe disability rather than achieving functional independence. This pattern may reflect the largely irreversible nature of the initial white matter injury caused by thalamic hemorrhage.

Regarding the optimal timing of intervention, our study observed a median time from admission to surgery of 8 h (IQR: 7–11.5). This timing reflects a strategic balance between the need for rapid decompression and the risk of postoperative rebleeding. While ultra-early hematoma evacuation (e.g., < 6 h from onset) is theoretically appealing for minimizing toxic injury, recent evidence suggests it may be associated with a higher risk of rebleeding due to the instability of the ruptured vessel during the hyperacute phase [25]. Our institutional protocol generally targets the 6–24 h window, aiming to intervene after the peak window of active hematoma expansion has passed but before irreversible secondary injury fully develops. We believe this strategy contributed to the favorable survival outcomes observed in the MIS group.

Our study has several limitations that imply caution in interpreting the results. First, as a retrospective, single-center study, our findings may lack external validity and may not be generalizable to institutions with different surgical protocols or patient demographics. Second, although PSM was used to balance baseline characteristics, selection bias cannot be entirely eliminated. Specifically, while we strictly excluded patients with pre-existing disability or coagulopathy, other potential confounders such as thalamic subregion involvement or precise white matter tract integrity were not explicitly stratified due to sample size constraints. Third, standardized 24-hour follow-up CT scans were not available for all patients, particularly those with stable clinical courses, preventing a systematic analysis of hematoma expansion rates between groups. Fourth, data regarding Withdrawal of Life-Sustaining Therapy (WLST) or Do Not Resuscitate (DNR) orders were not explicitly quantified. We acknowledge that variations in end-of-life care decisions can influence mortality rates. However, all patients included in the conservative group received active medical management based on AHA/ASA guidelines rather than palliative care on admission. Furthermore, since the primary clinical drivers of WLST—specifically deep coma (GCS score) and hematoma volume—were strictly matched between the two groups, the potential bias arising from differential withdrawal of care is mitigated in this comparative analysis. Fifth, the sample size in the matched cohort (*n* = 102) was relatively small. This limited statistical power may have prevented the detection of modest benefits in functional outcome (mRS 0–2) for the minimally invasive surgery group. Sixth, while our prognostic models demonstrated good internal discrimination, they lack external validation on an independent cohort. Finally, our outcome assessment was limited to 3 months. Given that recovery from thalamic hemorrhage can be prolonged, longer-term follow-up (e.g., 6 to 12 months) represents a critical direction for future research.

## Conclusion

In conclusion, our study suggests that age, SBP, and HV are independent predictors of poor prognosis in patients with TH, while a higher GCS score is protective. We developed and validated a nomogram to support individualized risk stratification. Notably, we identified distinct HV thresholds: HV > 7 mL was associated with poor functional outcome, whereas HV > 13 mL was associated with mortality. Based on these cutoffs, in the propensity score–matched analysis of patients with HV ≥ 7 mL, MIS was associated with a lower 90-day mortality rate compared with conservative treatment. However, MIS was not associated with a higher rate of functional independence (mRS 0–2) in this cohort. These findings suggest MIS may be associated with survival benefits in patients with HV ≥ 7 mL. Further studies are needed to optimize strategies for functional recovery, and large-scale, multicenter randomized controlled trials are warranted to validate these thresholds and refine surgical indications.

## Supplementary Information

Supplementary Material 1.

Supplementary Material 2.

Supplementary Material 3.

Supplementary Material 4.

## Data Availability

Data are available from the corresponding author upon reasonable request and with institutional approval.

## Abbreviations

ANOVA: Analysis of variance
AUC: Area under the curve
BP: Blood pressure
CI: Confidence interval
CT: Computed tomography
DBP: Diastolic pressure
GCS: Glasgow Coma Scale
HV: Hematoma volume
ICH: Intracerebral hemorrhage
IQR: Interquartile range
MIS: Minimally invasive surgery
mRS: Modified Rankin Scale
NES: Neuroendoscopic-assisted hematoma evacuation surgery
OR: Odds ratio
PSM: Propensity Score Matching
ROC: Receiver operating characteristic
SD: Standard deviation
SBP: Systolic blood pressure
SHE: Stereotactic Hematoma Evacuation
TH: Thalamic hemorrhage
