# Supplementary material for: Optimal hematoma volume cutoffs and efficacy of minimally invasive surgery for thalamic hemorrhage: a propensity score-matched analysis
Source: BMC Neurol. 2026 Feb 27;26:217. doi: 10.1186/s12883-026-04748-1 (PMC13049946; doi:10.1186/s12883-026-04748-1)
Supplement: Supplementary file 2 — Supplementary Material 2. [file 12883_2026_4748_MOESM2_ESM.docx]

| **Supplementary Table 1. Univariate logistic regression analyses for poor 3-month functional outcome** | | | | | |
| --- | --- | --- | --- | --- | --- |
| **Variables** | β | S.E | Z | *P* | OR (95%CI) |
| Age | 0.04 | 0.01 | 3.01 | 0.003 | 1.04 (1.02 ~ 1.08) |
| Sex |  |  |  |  |  |
| 0 |  |  |  |  | 1.00 (Reference) |
| 1 | -0.48 | 0.27 | -1.80 | 0.07 | 0.62 (0.36 ~ 1.05) |
| Smoking history |  |  |  |  |  |
| 0 |  |  |  |  | 1.00 (Reference) |
| 1 | 0.00 | 0.27 | 0.00 | 1.000 | 1.00 (0.59 ~ 1.70) |
| Alcohol consumption |  |  |  |  |  |
| 0 |  |  |  |  | 1.00 (Reference) |
| 1 | 0.14 | 0.27 | 0.51 | 0.61 | 1.15 (0.67 ~ 1.96) |
| Hypertension |  |  |  |  |  |
| 0 |  |  |  |  | 1.00 (Reference) |
| 1 | 0.08 | 0.34 | 0.24 | 0.81 | 1.08 (0.56 ~ 2.10) |
| Diabetes |  |  |  |  |  |
| 0 |  |  |  |  | 1.00 (Reference) |
| 1 | 0.14 | 0.41 | 0.36 | 0.72 | 1.16 (0.52 ~ 2.56) |
| SBP | 0.02 | 0.01 | 3.37 | <.001 | 1.02 (1.01 ~ 1.03) |
| DBP | 0.01 | 0.01 | 0.80 | 0.43 | 1.01 (0.99 ~ 1.02) |
| NHISS | 0.23 | 0.03 | 7.68 | <.001 | 1.26 (1.19 ~ 1.34) |
| GCS | -0.47 | 0.07 | -6.72 | <.001 | 0.63 (0.55 ~ 0.72) |
| IVH |  |  |  |  |  |
| 0 |  |  |  |  | 1.00 (Reference) |
| 1 | 1.14 | 0.30 | 3.75 | <.001 | 3.12 (1.72 ~ 5.64) |
| HV | 0.37 | 0.05 | 7.47 | <.001 | 1.44 (1.31 ~ 1.59) |
| Graeb score | 0.30 | 0.07 | 4.51 | <.001 | 1.35 (1.18 ~ 1.53) |

OR: Odds Ratio, CI: Confidence Interval.Reference categories: Sex (female as reference), IVH (absent as reference), smoking/alcohol/hypertension/diabetes (no as reference).NIHSS was assessed in univariate analysis but excluded from the multivariable model due to collinearity with GCS.
