# Supplementary material for: Optimal hematoma volume cutoffs and efficacy of minimally invasive surgery for thalamic hemorrhage: a propensity score-matched analysis
Source: BMC Neurol. 2026 Feb 27;26:217. doi: 10.1186/s12883-026-04748-1 (PMC13049946; doi:10.1186/s12883-026-04748-1)
Supplement: Supplementary file 3 — Supplementary Material 3. [file 12883_2026_4748_MOESM3_ESM.docx]

**Supplementary** **Table 2. Multivariate logistic regression analysis of factors associated with poor 3-month outcome.**

| **Variables** | **β** | **S.E** | **Z** | ***P*** | **OR (95%CI)** |
| --- | --- | --- | --- | --- | --- |
|  |  |  |  |  |  |
| Age | 0.07 | 0.02 | 3.58 | <.001 | 1.08 (1.03 ~ 1.12) |
| SBP | 0.01 | 0.01 | 2.04 | 0.04 | 1.01 (1.01 ~ 1.03) |
| GCS | -0.24 | 0.08 | -3.00 | 0.003 | 0.78 (0.67 ~ 0.92) |
| HV | 0.35 | 0.06 | 5.74 | <.001 | 1.42 (1.26 ~ 1.59) |
| Graeb Score | 0.10 | 0.10 | 1.00 | 0.32 | 1.10 (0.91 ~ 1.34) |

OR , Odds Ratio; CI, Confidence Interval; SBP, Systolic Blood Pressure; GCS, Glasgow Coma Scale; HV, Hematoma Volume.
