# Supplementary material for: Optimal hematoma volume cutoffs and efficacy of minimally invasive surgery for thalamic hemorrhage: a propensity score-matched analysis
Source: BMC Neurol. 2026 Feb 27;26:217. doi: 10.1186/s12883-026-04748-1 (PMC13049946; doi:10.1186/s12883-026-04748-1)
Supplement: Supplementary file 4 — Supplementary Material 4. [file 12883_2026_4748_MOESM4_ESM.docx]

| **Supplementary Table 3. Baseline hematoma volume specific cutoff values in patients with TH** | | | | |
| --- | --- | --- | --- | --- |
| **Prognosis** | **Cutoff volume** | **AUC** | **Sensitivity** | **Specificity** |
| Good | ＜7.0 | 0.86 | 0.75 | 0.83 |
| Mortality | ＞13.0 | 0.81 | 0.65 | 0.95 |

HV, Hematoma volume; AUC, Area under the curve; TH, thalamic hemorrhage
